# Joint developmental trajectories of internalising and externalising behaviours from childhood to adolescence and their links to socio-economic profiles - findings from the ‘growing up in Ireland’ cohort

**DOI:** 10.1007/s00787-025-02814-6

**Published:** 2025-07-10

**Authors:** Frances M. Cronin, Valéria Lima Passos, Debbi Stanistreet, Richard Layte

**Affiliations:** 1 School of Population Health, Royal College of Surgeons in Ireland, Dublin, Ireland; 2 School of Pharmacy and Biomolecular Sciences, Royal College of Surgeons in Ireland, Dublin, Ireland; 3 Present Address: Department of Sociology, Trinity College Dublin, Dublin, Ireland

**Keywords:** Internalising problems, Externalising problems, Children, Adolescent, Mental health, Group-based-multi-trajectory model

## Abstract

**Introduction:**

Compared to their more affluent peers, children of low socioeconomic position (SEP) are more likely to experience poor mental health. Correctly identifying factors that influence internalising and externalising symptoms is imperative for early, targeted intervention. Distinguishing between symptoms that are transient and chronic, and singular or co-morbid is important. We investigated how internalising and externalising behaviours co-occur in children from childhood to adolescence, and examined the association between these behaviours and childhood family- and child-level risk factors - including maternal characteristics, economic strain and experiences in childhood.

**Method:**

Using the large, nationally representative, longitudinal sample from the Growing Up in Ireland study, two mixture modelling techniques were applied: Latent Class Analysis to unveil multidimensional family profiles by clustering subjects with similar familial, maternal and lived experiences, and Group-Based Multi-Trajectory Model to identify joint behavioural trajectories based on responses to the internalising and externalising subscales of the Strength and Difficulty Questionnaire, measured at three time points (age 9, 13 and 17).

**Outcome:**

Half of the children (51%) showed no behavioural problems, however a large proportion of children experienced substantial economic strain while growing up in Ireland during the 2000s. Worse SEP conditions were linked to less advantageous maternal characteristics, e.g. mothers who were young, smoked and did not breastfeed. A clear gradient of troubled behaviour was seen as socio-economic difficulties increased. Changes in family profiles were associated with minor or modest alterations in behavioural outcomes. Implications for policy are discussed.

**Supplementary Information:**

The online version contains supplementary material available at 10.1007/s00787-025-02814-6.

## Introduction

Compared to their more affluent peers, children of low socioeconomic position (SEP) are more likely to experience adverse health outcomes [1–3], are at higher risk of experiencing mental health difficulties both immediately and in the long-term, and are two to three times more likely to be diagnosed with adult mental health problems [2, 4].

Elevated behaviour scores in childhood are consistently associated with adverse outcomes in adulthood in terms of personal, health and economic status [5–7]. Longitudinal studies show that high scores for externalizing behaviours (e.g. oppositional defiance disorder, conduct disorder and hyperactivity/inattention disorders [8]) in children are associated with later academic failure, delinquent behaviour, and criminality and/or violence [6, 9–11]. Elevated rates of internalizing behaviours (e.g. problems with peer relationships and emotional difficulties) in children are associated with depression and anxiety in adulthood [12].

Correctly identifying factors that influence internalising and externalising symptoms is imperative for early, targeted intervention, improving not only the well-being of the child or adolescent, but also their later adult health outcomes and life chances [13]. Distinguishing between symptoms that are transient and chronic, and singular or co-morbid, is important in order to consider the treatment or intervention [14]. This is particularly true during adolescence, a developmental period marked by rapid changes in physical, personal and social dimensions, with shifts from dependency on parents to personal autonomy, a change in the maturing child’s social circle, and an increase in their autonomy in choice and decision making [15].

Up to a recent past, many studies examined internalising and externalising difficulties in isolation, and therefore overlooked any temporal pattern of association between them [16]. In a longitudinal context, a commonly used approach has been to examine incidences of one of the difficulties separately, and cross-link their frequencies of occurrence. For example, Nivard et al. (2017) [14] conducted univariate modelling on internalising and externalising symptoms separately and investigated their cross-linkages. They concluded that the developmental trajectories of internalising and externalising symptoms were similar to each other and associated: e.g. high externalising was associated with high internalising behaviour [14]. To date, empirical evidence stemming from studies modelling the behavioural symptoms simultaneously suggest that they are neither mutually exclusive nor totally independent from each other [14, 17–20]. It is therefore important to further examine how these two diagnostic groups of disorders co-occur and/or jointly progress in order to inform effective treatments and interventions [21].

### Person-centred approach

Person-centred approaches, of which univariate and multivariate trajectory models are examples [22–24], are used to provide a more nuanced depiction of the complex realities of the course of development. Among others, they have been considered to address questions of whether and how different risks and protective factors manifest themselves in an individual child in different ways – specifically in terms of rate or magnitude of change [25].

Similar to the methodology employed by Girard (2021), who worked with the younger cohort of the Growing Up in Ireland (GUI) study, we employed 11 factors to broadly represent the risk and protective factors described in the ecological systems theory of development [18, 26]. These factors have been associated with behavioural outcomes in childhood and adolescence, and include: duration of breastfeeding [27–30]); maternal smoking during pregnancy [31, 32]); maternal depression [33]; maternal age [34, 35]; familial financial hardship, or economic strain (considered to predict onset of all classes of disorders at every life-course stage, but to be unconnected to the severity or persistence of the disorder [4, 36]). Finally, maternal perception of the family neighbourhood safety was included as it has been identified as a factor of influence in mental health outcomes for children [37–39].

### Aims of study

1. To identify joint trajectories of internalising and externalising behaviours from childhood through to young adulthood;
2. To examine the association between these trajectories and distinct family profiles (defined by combinations of maternal and socio-economic time-stable and time-varying factors) in order to assess whether (and how) the profiles differentiate children with behavioural difficulties from those with low or no reported difficulties.

To the best of our knowledge this is the first study in Ireland to use a large, nationally representative, longitudinal sample to investigate the co-occurrence of externalising and internalising behaviour during childhood through adolescence using a person-centred approach.

## Methods

### Study design and population

This study uses observational data on young people from Cohort 98, one of the two cohorts that form the Growing Up in Ireland Study (GUI), a nationally representative, longitudinal cohort of young people from Ireland (Williams et al. 2009). Cohort 98 was first sampled age 9 in 2007, and subsequently followed up age 13 in 2011, age 17 in 2016 and age 20 in 2019. Baseline data collection in 2007 sampled 8568 9-year-old children clustered in 910 primary schools in the Republic of Ireland. Wave 2, conducted 4 years later at age 13, retained 88% (*N* = 7525) of the initial sample. Wave 3, at age 17/18, retained 72.5% of the original sample (*N* = 6216).

The data were reweighted (Murphy et al. 2018; Thornton et al. 2010) for analyses to take account of the original sample error and subsequent attrition using a range of factors and a minimum distance algorithm. The final reweighted sample is representative of young people who were residing in Ireland at 9 years of age in 2006, and who continued to live in Ireland at 17/18 years of age in 2016.

Further details on the design and reweighting procedures are available elsewhere (Murphy et al. 2018; Thornton et al. 2010).

### Outcome variables

#### Internalising and externalising symptom scores

Behaviour problems at 9, 13 and 17 were assessed using the mother’s rating of the child’s behaviour on the Strengths and Difficulties Questionnaire (SDQ, Goodman 1997 [40]). The SDQ is a well-validated, widely used instrument that is used to assess the mental health of children and adolescents aged 6–18 years both cross-sectionally and over time [41]. In the GUI dataset, the internal consistency of the Total Difficulty (TD) score for age 9 (α coefficient = 0.77; [42]) age 13 (0.85) and age 17 (0.83) were in line with Goodman (0.82) [43]. The individual (five) subscales of the SDQ is more suited for use with high-risk children [44], therefore, the broader scales of Internalising (sum of the emotional and peer problems subscales, score range 0–20,) and Externalising (sum of conduct problems and hyperactivity-inattention subscales, score range 0–20) were employed. These two scales are more convergent, have discriminant validity in low-risk samples, and differentiate between different risk groups [45]. SDQ cut-off points identified approximately 20% of children with raised and/or high scores for internalising (3+) and externalising (7+) behaviours [45].

### Risk factors

#### Family profiles

Breastfeeding was dichotomised into ‘breastfed for 4 months+’ yes/no. Center for Epidemiological Studies Depression Scales (CES-D)^1^ [46], measured mothers’ symptoms of depression in the previous week (range 0–24, (1 = depressed (score of 7+), 0 = not depressed). At each wave, mothers indicated the degree of ease or difficulty they had in making ends meet^2^. Economic strain was dichotomised (1 = ‘with great difficulty’, ‘with difficulty’, 0 = ‘with some difficulty’, ‘fairly easily’, ‘easily’, ‘very easily’). Mothers’ perceptions of their neighbourhood identified if the child could ‘play safely’ (age 9), or could safely ‘hang out’ (age 13 & 17) (Yes/No). Measured at baseline, maternal education was categorised: 1 = Degree level or higher, 0 = Less than degree level.

The effect of the 2008 Irish recession on the family (See Appendix 1) was categorised (1 = ‘very significant effect’ and ‘significant effect’ and ‘small effect’, 0 = ‘no effect’ and ‘don’t know’). Mother’s age at birth of the child was calculated as mother’s age less child’s age at baseline. Mothers were asked if they smoked while pregnant (Yes/No).

### Statistical analyses

All measures and variables used in the study, together with responses and wave in which they were measured, are reported in Online Resource 1, Appendix 2, Table 1.

#### Analysis plan

Firstly, the selected risk factors were amalgamated by means of Latent Class Analysis (LCA) to capture distinct combinations of a child’s family factors (broadly in terms of their mother’s features and socioeconomic environment). Second, in keeping with a person-centred approach, the patterns of co-evolution of internalising and externalising behaviours over time was examined using Group-Based Multi-Trajectory Modelling (GBMTM [22–24]), in order to explore the heterogeneity of their temporal cross-linkages in youth. Finally, associations between the family profiles and the internalising and externalising trajectory outcomes were explored.

LCA is used to address heterogeneity of answers to categorical questions by grouping subjects displaying similar scoring patterns [47, 48]. Herein, LCA was employed to derive latent subgroups, or ‘Family profiles (Fam. Prof)’ incorporating multidimensional features of related mothers’ and children’s characteristics: maternal education, age, prenatal smoking, depression and breastfeeding practices, indicators of economic strain, neighbourhood unsafety and 2008 economic recession impact. GBMTM is applied for the recognition and visualisation of different patterns of temporal change, e.g. typical or atypical courses of development of one or multiple outcomes [22]. In this study, GBMTM was applied to obtain data-driven, temporally dynamic classes of internalising and externalising co-development of behaviours over time. The analyses unfolded step-wise: firstly, the class-enumeration was established for LCA and GBMTM separately; and second, identified multivariate trajectories were regressed on the extracted family profile latent classes.

#### Model selection - latent class analysis and group-based multivariate trajectory models

Model fitting for LCA and GBMTM was based on statistical model-fit criteria, e.g. Akaike Information Criterion (AIC) and Bayesian Information Criterion (BIC). Entropy was used as an index for model performance with regards to classification uncertainty. Interpretability of extracted classes and their visual distinctiveness were factored in when selecting the final number of classes/trajectories [49]. Once these were established, post-hoc posterior probabilities of class-membership were estimated for each individual, who was then assigned to the class for which they had the highest posterior probability of assignment (PPA) - modal assignment. Online Resource Appendix 3, Table 2 displays the values of model fit criteria used to guide the choice of the final number of classes in LCA and also describes similar technical elements of the GBMTM’s class-enumeration.

Lastly, multinomial logistic regression was used to link the family LCA profiles to the multivariate GBMTM trajectories. To account for the uncertainty of class assignment, the subjects’ class-membership probabilities, i.e. their posterior probabilities of assignment (LCA-PPAs), instead of the classes themselves, were considered the independent variable in the multinomial logistic model.

## Results

### Family profiles – LCA results

LCA models with 2 to 8 latent classes were explored. A 6-class solution offered a good fit to the data and classification uncertainty was accounted for by using the subjects’ class-membership probabilities (i.e. their posterior probabilities of assignment (LCA-PPAs)), instead of their final classification, as independent variables in the multinomial logistic regression model. Technical details of the model fit and selection are provided in Appendix 2.

**Fig. 1:**
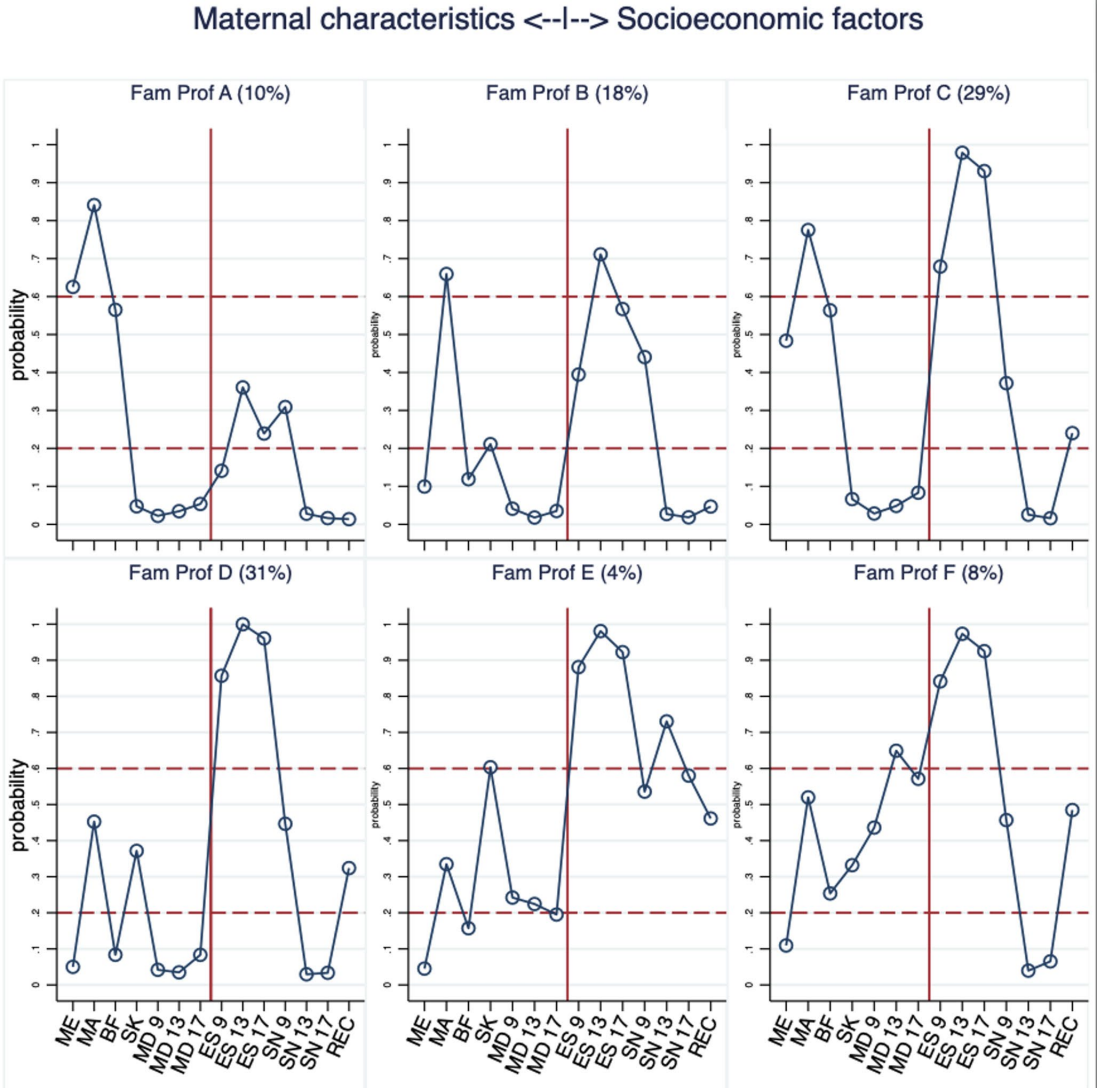
LCA’s Item Probability Plot with the 6 extracted family profiles, based on maternal and socioeconomic indicators. KEY: Probability: High (60%+), Mid (> 20–59%) Low ( < = 20%). ABREVIATIONS: ME maternal education, **Degree+**; MA Mother’s age, **35 + years**; BF breastfeeding, **4 months+;** SK prenatal smoking, **Yes**; MD maternal depression, **Yes**; ES economic strain **High**; SN Safe neighbourhood, **No**; REC impact of recession, **High**. **Fam Prof A** ‘Comfortable’ **Fam Prof B** ‘Older mother, low education, low breastfeeding’ **Fam Prof C** ‘Older mother, medium education’ **Fam Prof D** ‘Challenged young mother’ **Fam Prof E** ‘Young mother, low depression, feel unsafe’ **Fam Prof F** ‘Most vulnerable’

Six ‘Family Profiles’ (Fam Prof, latent classes) were extracted, each depicting distinct cross-combinations of maternal and socio-economic characteristics, unveiling different patterns of their co-occurrence and their prevalence. Further, because some of the indicators were repeatedly measured over time, the family profiles capture also temporal dynamics, allowing for a glimpse into the pre (age 9), during (age 13) and post (age 17) 2008 recession changes. Figure 1 displays the item probability plot showing the probability of the subjects assigned to each of the latent classes to have selected the indicated categories of these maternal and socio-economic indices. In general, substantial variability was observed in indices related to economic strain, recession impact and maternal features, with clear differences among the extracted classes. By contrast, the prevalence of maternal depression was low (for most profiles), with only one group showing a relatively elevated risk over the follow-up years. Similarly, in this population neighbourhood was deemed safe for most children, undergoing a substantive drop (improvement during and after age 13). Specifically, in family profile A (Fam Prof A, ‘Comfortable’ (10%)), were children born to mothers who were older, had been educated to degree level or above and exercised beneficial health behaviours around pregnancy (low probability of smoking, and high probability of breastfeeding for 4 months or longer). Fam Prof A mothers were also unlikely to report depression and to consider their neighbourhoods safe. In comparison, the most vulnerable group identified (Fam Prof F, ‘Most vulnerable’, 8%) consisted of children of the youngest mothers who reported depression, the highest economic strain at all time-points and the highest probability of a high impact of recession. The largest group of children (Fam Prof D, ‘Challenged young mother’ 31%) had mothers who had less than degree level education, were likely smokers while pregnant and did not breastfeed. Despite the high economic strain reported by these mothers, they experienced no depression.

Older mothers were associated with Fam Prof A, Fam Prof B (‘Older mother, low education, low breastfeeding’, 18%) and Fam Prof C (‘Older mother, medium education’, 29%). These family profiles varied in maternal education (high, low and medium probability of degree level education respectively). While maternal depression was not a conspicuous feature of these particular family profiles, Fam Prof B had a lower breastfeeding prevalence compared with Fam Profs A and C.

There was a significant change for each profile in neighbourhood safety rating from relative high probability of feeling unsafe at age 9 to a lower probability of a negative rating at ages 13 and 17. This was consistent across all profiles apart from Fam Prof E (4%, ‘Young mother, low depression, feel unsafe’), which, compared to all profiles, rated their neighbourhood as unsafe at all times.

For all profiles, the single item ‘impact of the recession’ mirrored the relatively higher ‘economic strain’ at age 13 compared to age 9 and 17. Although economic strain was elevated for all profiles, this burden was observed to a lesser degree in the more advantageous profiles (A and B).

.

### Internalising and externalising joint trajectories – GBMTM results

**Fig. 2:**
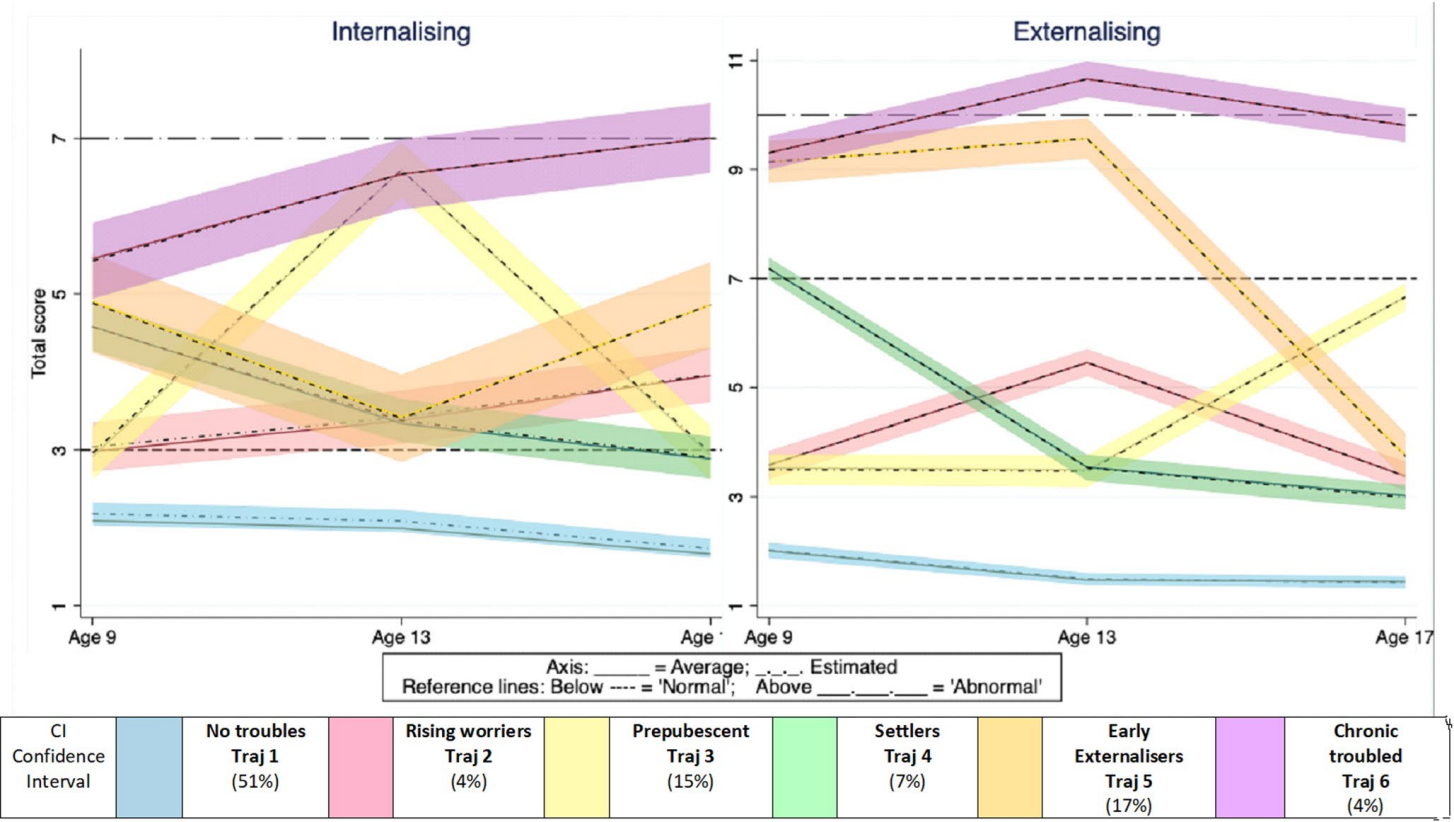
Joint developmental trajectories of internalising and externalising behaviours together with estimated line and 95% confidence bands

Six distinct concomitant trajectories of internalising/externalising behaviours were identified, depicting patterns of symptom co-occurrence (Fig. 2). Above normal internalising scores were the most prevalent feature, having been observed in 5 out of 6 trajectories.

Just over 50% of the children showed no behavioural difficulties (Trajectory (Traj 1), blue, 51%) with ‘No troubles’ in either domain. Traj 4 (‘Settlers’, green, 7%,) had above average internalising scores, trending downward to ‘normal’ over time, with abnormal externalising at age 9 only, reducing to low at 13 and normal at 17.

The smallest group was the most problematic (Traj 6, ‘Chronic troubled’, purple, 4%). For this group, internalising was ‘high’ and increased over time, while externalising raised from borderline (high ‘above normal’) to ‘abnormal’ at 13 and settled at borderline at 17. The second largest group (Traj 5, ‘Early externalisers’, orange, 17%) reported above normal internalising scores at all time-points, with borderline externalising (high ‘above normal’) at ages 9 and 13 only, dropping dramatically to normal scores at age 17.

Traj 3 (‘Prepubescent’, yellow, 15%), had low internalising and externalising levels at all ages, except for a spike of internalising difficulties at age 13. Lastly, Traj 2 (‘Rising worriers’, red, 4%) had low but trending higher internalising, and raised but normal externalising values at 13.

### Association between family profile and behaviour trajectories, adjusted for sex

**Fig. 3:**
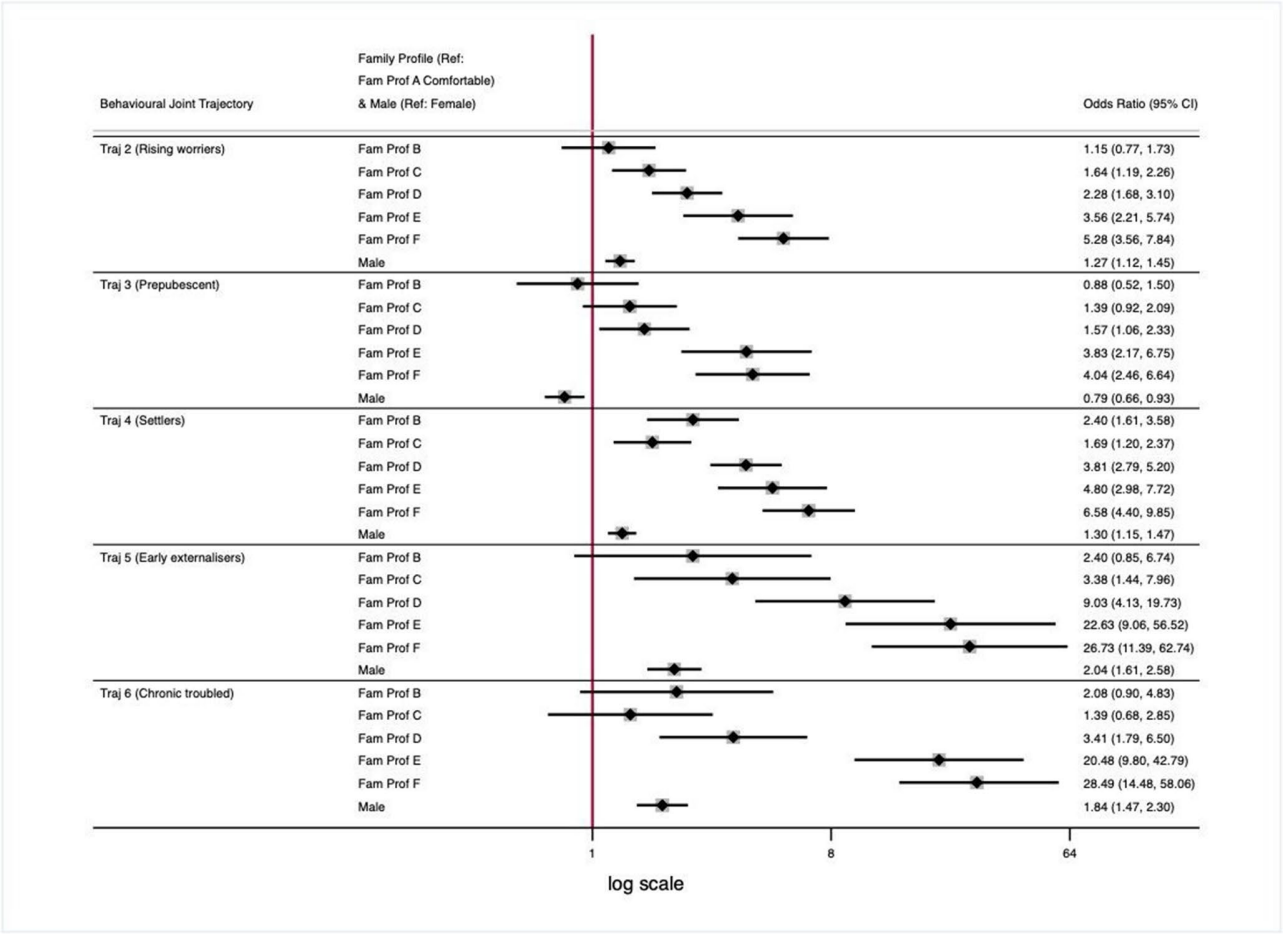
Forest plot of associations between extracted LCA (family profiles) and GBMTM joint behaviour trajectories, adjusted for sex

After adjustment for sex, a family profile gradient was detectable both within and across trajectories (Fig. 3). For example, using Fam Prof A ‘Comfortable’ as a reference, the odds for being a member of Traj 2 (Rising worriers) relative to Traj 1 (No troubles) (first row forest plot) gradually increased from the Fam Prof B through to Fam Prof F, i.e. from the less- to the more- socio-economic disadvantaged family profiles. This gradient manifested itself also in the other trajectories (additional forest plot rows), increasing in magnitude of effect size (ORs), moving from the less- to the more- problematic behavioural trajectories (Traj 2 to 6). In addition, the graded association seemed interrupted in Traj 4 (Settlers), specifically when comparing the ORs between Fam Prof B and C: members of Fam Prof B (‘Older mother, low education, low breastfeeding’), instead of Fam Prof C (Older mother, medium education’), had slightly higher odds to be assigned to this ‘Settlers’ trajectory compared to the ‘no troubles’. While Fam Prof C is more socio-economically challenged compared to Fam prof B, the maternal variables are better: mothers from Fam Prof C have higher education, are a little older, have breastfed their children and have low probability of being a smoker. These results suggest that the more advantageous maternal profile might better counteract the socio-economic adversity.

Males were more likely to have been assigned to all trajectories compared to the Traj 1 (No troubles), except for Traj 3 (Prepubescent), in which a stronger female representation was observed. This trajectory was characterised by internalising issues only (high level at age 13), but normal externalising values, with a slight elevation from ages 13 to 17.

## Discussion

The aim of this research was to investigate how internalising and externalising behaviours co-occur as children mature from childhood to adolescence, and to examine the association between these behaviours and childhood family- and child-level risk factors - including maternal characteristics, economic strain and experiences in childhood.

To the best of our knowledge this is the first study in Ireland to investigate patterns of joint externalising and internalising behaviours during childhood through adolescence using a person-centred approach.

We identified six distinct family profiles, and six behavioural trajectories. When linking profiles with trajectories, a clear gradient of troubled behaviour was seen as socioeconomic and economic difficulties increased. Socio-economic differentials were seen in all comparisons, indicating that changes in socio-economic and/or family experiences were associated with minor or modest alteration in behavioural outcomes. While just under 50% of our sample exhibited some combination of externalising and/or internalising difficulties, 4% exhibited co-occurring chronic difficulties (Traj 6 – Chronic Troubled).

Every family profile was featured in every behaviour trajectory (Online Resource Appendix 4, Table 3). ‘Chronic troubled’ trajectories were present in the Comfortable family profile, as well as the Vulnerable. However, when adjusted for sex, the most challenged trajectory of ‘Chronic troubled’ were those raised in two practically identical family profiles (each sharing low maternal education, low-mid probability of breastfeeding, high economic strain) that differed primarily in relation to maternal depression. This finding is of interest, especially in light of the recent meta-analytic review [33] that examined the strength of association between maternal depression and adverse child outcomes including internalising and externalising problems. The review (*n* = 193 studies) concluded that of the five child outcomes (internalising/externalising behaviour, general psychopathology, and negative/positive affect), most of the variance was not accounted for by maternal depression even when moderators were taken into account. Furthermore, maternal depression was no more strongly associated with internalizing than with externalizing problems. The review underscored the importance of recognising the cumulative or interacting effects that are likely to be the most accurate predictors of child outcomes, and called for developing and testing models with multiple co-occurring risk factors [33].

The ‘Prepubescent’ trajectory consisted primarily of females, and were children of Fam Profs that shared elevated rates of maternal depression and high economic strain at all time points, reflecting the contention that economic strain results in a disproportionately heavy burden on females (both mothers and girls) during times of recession [50]. Notwithstanding the ‘Prepubscent’ trajectory, our results found males to be at greater risk than females for membership of all troubled behavioural trajectories. This included being twice as likely to be assigned to the ‘Early externalisers’ trajectory where high internalising at 9 and 13 as dramatically reduced at age 17 when externalising symptoms increased sharply. Whether the spike in externalising behaviour is a response of the earlier internalising difficulties might be a question for future analysis.

Unsurprisingly, family profiles with the poorest maternal and socio-economic characteristics were associated with more detrimental outcomes at all ages, with the most extreme conditions being linked to chronically poor behaviour in both internal and externalising variables. The family profiles consisting of older mothers, low rates of maternal depression and differentiated by high maternal education and low economic stress had children with similarly low or no probability of being members of ‘Prepubescent’ or ‘Chronic troubled’ trajectories.

Of interest is the comparison of our results with those using the GUI Infant Cohort (Irish children followed from ages 3 through 9) [18]. Both studies found that low maternal age did not increase the risk of membership of the most chronically troubled group - our most challenged profile had over 50% probability of the mother being aged 35+. In addition, despite only 24% of the infant cohort exhibiting elevated levels of internalising, externalising and/or peer problems, the proportion suffering chronic elevated problems was comparable: 3% of the 3–9 year olds reporting chronic difficulties [18] compared to 4% of 9–17 year olds of this study (Traj 6). Future sweeps of the GUI Infant Cohort might examine this troubled 3% to track behaviour trajectories from infancy through their adolescence and into their adulthood.

Our findings support the previously described association between socio-economic status, and behavioural outcomes [18, 51, 52]. Mothers in the most vulnerable Fam Prof (F) were unlikely to be educated to degree level, and were at the greatest risk of being depressed at all time points, particularly during the period of greatest economic strain. Furthermore, there was evidence that a more advantageous maternal profile counteracts socio-economic adversity. Fam Prof C, compared with Fam Prof B, was more socio-economically disadvantaged, but had better educated mothers, who had a high probability of breastfeeding and low probability of prenatal smoking. These children had higher odds of being assigned to Traj 4 (Settlers), compared to the ‘no troubles’, than the more socio-economically advantageous Fam Prof B.

Of note, and irrespective of behavioural outcome, our findings showed approximately 70% of this nationally representative analytical sample (Fam Profs C to F) are being raised in suboptimal circumstances - particularly in relation to economic strain. These suboptimal experiences cannot be conducive to a happy home life and the results highlight the necessity to focus supports on families in lower socioeconomic groups - particularly and especially during down-turns in the general economy.

### Strengths and limitations

The strength of this study lies in the fact it is the first study in Ireland to use a large, nationally representative, longitudinal sample to investigate the co-occurrence of externalising and internalising behaviour during childhood through adolescence using a person-centred approach. In estimating exposure to risks, many comparable studies employ a summative overall score, giving equal weight to all variables. By our use of LCA to score risk factors, the relative and independent contributions of cumulative risk is accounted for [53].

Despite this, some limitations should be noted. As with many studies in this area, maternal reports were used to determine child behaviour, associated risks and protective factors together with perinatal and familial circumstances. By virtue of this fact, potential response biases, including recall and social desirability bias, may have been introduced. Furthermore, it should be noted that parental reports may be under-representing difficulties within this cohort. A study of over 24 countries found that when self-reported, adolescents reported significantly more problems than their parents reported about them [54].

The patterns of change transpired by the behavioural trajectories spanned critical years in the children-adolescence transition. As such, they conveyed a long-term behavioural picture during this formative period. However, despite the lengthy follow-up, the time scale’s granularity was coarse, having only three time points, with long intervals between them. Consequently, the identified behavioural paths captured rough general trends, missing nuances related to the degree of stability and/or fluctuations in internalising and externalising scores between the measured ages. Moreover, the three measurement points makes it difficult to establish whether any observed upward or downward shift in the trajectories was a random occurrence or the reflection of a meaningful peak/drop in the developmental process. In the tension between these two interpretational alternatives, long-term patterns of change vs. fluke oscillations, a cautionary posture is warranted. Nonetheless, despite these limitations, the graded alignment between the family profiles and the behavioural trajectories unveiled a cogent and theoretically meaningful link between underlying socio-economic processes and behavioural patterns.

## Conclusion

Disadvantaged families, by definition, lack access to resources including education, neighbourhood and physical environment, employment, social support networks, and access to health care. Socio-economically deprived children are “at risk of being at risk in the first place” [51, 52]. Some findings suggest that youth mental health promotion, prevention and early interventions should involve mental, primary and social care systems [55]. Others acknowledge that one type of intervention focusing on a particular set of protective factors may be effective for one individual, but not necessarily for another [56]. Regardless of the approach, in order to identify the correct intervention and to maximise positive outcomes, it is imperative that behavioural difficulties are identified as singular or co-morbid, transient or chronic [14, 15, 21]. Furthermore, policy makers must understand that a child raised in socioeconomic adversity is at increased risk of experiencing mental health difficulties as they develop, and that elevated adverse behaviours in childhood map directly onto later adult health and are associated with poor academic outcomes, social exclusion, lack of job opportunities and relationship difficulties [1–3, 57, 58]. Unless specific and targeted interventions are put in place during economic downturns, these vulnerable children and families are disproportionately affected, and the difficulties for the child, the adult and society are exacerbated [2, 4].

## Electronic supplementary material

Below is the link to the electronic supplementary material.

Supplementary Material 1

## Data Availability

The datasets generated and/or analysed during the current study are available in the Irish Social Science Data Archive, University College Dublin, Ireland https://www.ucd.ie/issda/data/growingupinirelandgui/).

## Footnotes

1 https://www.growingup.ie/pubs/Summary-Guide_Child-Cohort_Wave-1-1.pdf.

2 MML2, Pc2g36 pc3g13 (Making ends meet).
