# Supplementary material for: Joint developmental trajectories of internalising and externalising behaviours from childhood to adolescence and their links to socio-economic profiles - findings from the ‘growing up in Ireland’ cohort
Source: Eur Child Adolesc Psychiatry. 2025 Jul 10;35(1):109–19. doi: 10.1007/s00787-025-02814-6 (PMC12917099; doi:10.1007/s00787-025-02814-6)
Supplement: Supplementary file 1 — Supplementary Material 1 [file 787_2025_2814_MOESM1_ESM.pdf]

Online Resource

SUPPLEMENTARY MATERIALS

Joint developmental trajectories of internalising and externalising behaviours from childhood to adolescence and their links to Socio-economic profiles - Findings from the 'Growing Up in Ireland' cohort.

Frances M. Cronin[1], Valeria Lima Passos[1], Debbi Stanistreet[1] & Richard Layte[2]

[1]Royal College Of Surgeons In Ireland, Dublin 2, Ireland

[2]Department of Sociology, Trinity College Dublin, Dublin, Ireland

Corresponding author: Valeria Lima Passos, Royal College of Surgeons in Ireland, Dublin 2, Ireland. Telephone: ++353 87 943 8685 valerialimapassos@rcsi.ie

Appendix 1

The baseline data for the Growing Up in Ireland Cohort 98 were collected over a six-month period in the second half of 2007 and first half of 2009 when the children were within a month of their ninth birthday.

On the morning of 30th September 2008 the people of Ireland woke to the news that overnight the Irish Government had issued a blanket guarantee of all deposits, senior unsecured debt, securities and dated subordinated debt in Irish banks. The housing market had been slowly falling for a year and unemployment had been rising for nine months but the bank guarantee was perceived as the official start of Ireland's financial crisis.

In the summer of 2009, Ireland was the first European country to enter recession and the following five years were marked by enormous hardship and financial stress across the country. Between 2009 and 2012 average household income fell by over 17% and unemployment tripled from 6% to 18%. The experience of recession among the Growing Up in Ireland cohort has been shown to be strongly associated with decreases in parental mental health as well as worsening emotional and behavioural problems (Richard Layte and McCrory 2018).

TABLE S1 Joint behaviour trajectories groups by demographic, maternal and behaviour characteristics

	Joint developmental trajectories of Internalising and Externalising behaviours							
	Traj 1	Traj 2	Traj 3	Traj 4	Traj 5	Traj 6		
	No troubles	Rising worriers	Prepubescen t	Settlers	Early externalisers	Chronic troubled	Total	
	4157 (51%)	1235 (15%)	626 (8%)	1387 (17%)	322 (4%)	361 (4%)	(N = 8088)	*p-value
**Mother education (Age9)**								<0.001
Non degree level	2933 (70.6%)	923 (74.7%)	449 (71.7%)	1109 (80.0%)	**272 (84.5%)**	301 (83.4%)	5987 (74.0%)	
Degree+	1224 **(29.4%)**	312 (25.3%)	177 (28.3%)	278 (20.0%)	50 (15.5%)	60 (16.6%)	2101 (26.0%)	
**Mother's age at birth**								<0.001
<30 yrs	1330 (32.0%)	498 (40.3%)	252 (40.3%)	569 (41.0%)	146 (45.3%)	**178 (49.3%)**	2973 (36.8%)	
>=30 yrs	**2827 (68.0%)**	737 (59.7%)	374 (59.7%)	818 (59.0%)	176 (54.7%)	183 (50.7%)	5115 (63.2%)	
**>=4mnth breastfeeding BIN**								<0.001
<4mnths	2767 (66.7%)	846 (68.6%)	433 (69.2%)	1027 (74.2%)	236 (73.8%)	**283 (78.6%)**	5592 (69.3%)	
>=4mnths	**1380 (33.3%)**	388 (31.4%)	193 (30.8%)	357 (25.8%)	84 (26.2%)	77 (21.4%)	2479 (30.7%)	
**Prenatal smoking**								<0.001
No	**3418 (82.2%)**	933 (75.5%)	479 (76.5%)	1005 (72.5%)	215 (66.8%)	244 (67.6%)	6294 (77.8%)	
Yes	738 (17.8%)	302 (24.5%)	147 (23.5%)	381 (27.5%)	**107 (33.2%)**	117 (32.4%)	1792 (22.2%)	
**Maternal Depression Status age 9**								<0.001
Not depressed	**3590 (93.9%)**	1041 (91.6%)	530 (92.2%)	1165 (90.3%)	254 (87.3%)	268 (81.0%)	6848 (92.0%)	
Depressed	232 (6.1%)	95 (8.4%)	45 (7.8%)	125 (9.7%)	37 (12.7%)	**63 (19.0%)**	597 (8.0%)	
**Maternal Depression Status age 13**								<0.001
Not depressed	**3061 (94.6%)**	1050 (87.5%)	528 (88.1%)	1186 (88.4%)	254 (81.2%)	252 (71.6%)	6331 (89.9%)	
Depressed	176 (5.4%)	150 (12.5%)	71 (11.9%)	156 (11.6%)	59 (18.8%)	**100 (28.4%)**	712 (10.1%)	
**Maternal Depression Status age 17**								<0.001
Not depressed	**2350 (92.1%)**	811 (85.5%)	500 (82.1%)	972 (89.1%)	206 (84.8%)	210 (74.7%)	5049 (88.2%)	
Depressed	201 (7.9%)	138 (14.5%)	109 (17.9%)	119 (10.9%)	37 (15.2%)	**71 (25.3%)**	675 (11.8%)	
**Economic Strain Age9**								<0.001
No econ strain	**1737 (41.8%)**	399 (32.4%)	230 (36.8%)	439 (31.7%)	70 (21.7%)	98 (27.1%)	2973 (36.8%)	
Economic strain	2416 (58.2%)	834 (67.6%)	395 (63.2%)	948 (68.3%)	**252 (78.3%)**	263 (72.9%)	5108 (63.2%)	
**Economic Strain Age13**								<0.001

No econ strain	**588 (18.0%)**	145 (11.9%)	78 (12.9%)	144 (10.6%)	16 (5.1%)	32 (9.1%)	1003 (14.1%)	
Economic strain	2673 (82.0%)	1070 (88.1%)	525 (87.1%)	1213 (89.4%)	**300 (94.9%)**	321 (90.9%)	6102 (85.9%)	
**Economic Strain Age17**								<0.001
No econ strain	**663 (25.6%)**	177 (18.4%)	117 (19.0%)	223 (20.1%)	42 (16.7%)	39 (13.6%)	1261 (21.7%)	
Economic strain	1926 (74.4%)	787 (81.6%)	498 (81.0%)	886 (79.9%)	210 (83.3%)	**248 (86.4%)**	4555 (78.3%)	
**Safe area Age 9**								0.006
Safe area	2479 (59.7%)	**745 (60.5%)**	335 (53.6%)	814 (58.8%)	178 (55.3%)	192 (53.2%)	4743 (58.7%)	
Not safe	1673 (40.3%)	486 (39.5%)	290 (46.4%)	571 (41.2%)	144 (44.7%)	**169 (46.8%)**	3333 (41.3%)	
**Safe area Age 13**								<0.001
Safe area	**3124 (95.9%)**	1119 (92.2%)	561 (93.0%)	1264 (93.2%)	275 (87.6%)	302 (85.8%)	6645 (93.6%)	
Not safe	133 (4.1%)	95 (7.8%)	42 (7.0%)	92 (6.8%)	39 (12.4%)	**50 (14.2%)**	451 (6.4%)	
**Safe area Age 17**								<0.001
Safe area	**2491 (96.3%)**	909 (94.4%)	567 (92.3%)	1052 (95.0%)	233 (93.2%)	261 (90.6%)	5513 (94.9%)	
Not safe	97 (3.7%)	54 (5.6%)	47 (7.7%)	55 (5.0%)	17 (6.8%)	**27 (9.4%)**	297 (5.1%)	
**Recession Impact**								<0.001
Low to mod	**3463 (83.3%)**	892 (72.2%)	457 (73.0%)	978 (70.5%)	199 (61.8%)	225 (62.3%)	6214 (76.8%)	
High	694 (16.7%)	343 (27.8%)	169 (27.0%)	409 (29.5%)	**123 (38.2%)**	136 (37.7%)	1874 (23.2%)	

*Continuous: Mean (Standard Deviation) test statistic: ANOVA Categorical: N (%) test statistic: Chi-square

Appendix 2

Table 2. LCA model fit criteria for increasing number of latent classes

Nr. of Classes	AIC	BIC	CAIC	Adj. BIC	Entropy
2	5540.06	5752.01	5781.0	5659.85	0.54
3	4548.12	4856.04	4900.04	4716.22	0.54
4	3993.78	4406.67	4465.67	4219.18	0.53
5	3734.58	4252.45	4326.45	4017.29	0.58
6	**3589.38**	**4212.21**	**4301.21**	**3929.39**	0.55
7	3536.65	4264.46	4368.46	3933.97	0.56
8	3496.00	4328.78	4447	3950.62	0.57

AIC, Akaike's information criterion, BIC, Bayesian information criterion, CAIC, consistent Akaike's information criterion and adjusted BIC.

LCA Model fitting & selection – Class-enumeration

Three out of the four model fit-criteria, except AIC, improved with an increasing number of latent classes up to the 6-class solution (Table 2). Of note, entropy, as an index for model performance with regards to classification uncertainty, has only an auxiliary role in class-enumeration. Entropy is bounded by 0 and 1, with higher values indicating better classification. A value around 0.6 is considered a moderate classification performance, and below 0.4 poor.

Once the final LCA model is selected (and also GBMTM – see later on), post hoc posterior probabilities of class-membership are estimated for each individual, who are then assigned to the class for which they had the highest posterior probability of assignment (PPA) - modal assignment. This disregards the probabilistic nature of class-assignment. However, because the final LCA model had only a moderate classification performance, we deemed relevant to incorporate this classification uncertainty while deriving the multinomial logistic regression model linking the developmental profiles (LCA classes) to the Internalising and externalising trajectories. To this end, the subjects' class-membership probabilities, i.e. their posterior probabilities of assignment (LCA-PPAs), instead of their final classification, were entered as independent variables in the multinomial logistic regression model.

Group-Based multi-Trajectory Model (GBMTM)

Model fitting & selection

First, the number of latent trajectory clusters was established ("class-enumeration") using a supportive R-code that compiles fit-indices for several models with different numbers of latent clusters (Fit-criteria Assessment Plots[1] – F-CAP – for details see below). For this purpose, models with increasing numbers of clusters were run (3 to 10), all with quadratic polynomials. After class-enumeration, the non-significant high-order polynomials were pruned (p>=0.05; p-values higher that 0.05 were occasionally accepted, once deletion of the higher order led to a substantial deterioration of BIC).

Longitudinal data without a natural starting point can be characterized by substantial onset variability. If this happens to be the dominating data feature, mixture models, of which GBTM is a case, may underperform in their trajectories' shape-detecting capabilities. As a result, data partitioning will be dominated by level, instead of shape, yielding flat, sparse, parallel trajectories, and, consequently, process heterogeneity in the longitudinal data may go undetected.

- *Ad hoc strategy to improve GBMTM's shape detectability:*

When level variability appears to be the dominating data feature, Heggeseth et al.[2] suggested removing level by subtracting the within-individual mean score from all within-individual observations. Our *ad hoc* strategy to circumvent the problem of sparse data partitioning involved two steps. Similarly to Heggeseth at al., as the first step, we determined the longitudinal shape of an outcome variable $\boldsymbol{Y}$ by centering the scores. The vector of subject specific scores $\boldsymbol{Y_i}$ were centered, i.e. the scores were subtracted from the individual's average over the time points ($\boldsymbol{\bar{y}_i} = \boldsymbol{t_i^{-1}} \sum_{t=1}^{t_i} \boldsymbol{y_i}$), previous to model fitting. Let $\boldsymbol{A_i} = \boldsymbol{I_{t_i}} - \boldsymbol{t_i^{-1}} \mathbf{1}_{t_i} \mathbf{1}_{t_i}^T$ be the centering matrix. The vector of centered scores for subject *i* were obtained by $\boldsymbol{C_i} = \boldsymbol{A_i Y_i}$. We centered both Internalising and Externalising scores. As the second step, different from Heggeseth et al., we then coupled centered values back to the original ones by using the multivariate version of GBTM. Thus, for these outcomes original and centered scores were included in the multivariate GBMTM (for both, the Censored Normal distribution was applied as link function).

Brief explanation of the FCAP:

The Fit-Criteria Assessment Plot (F-CAP) is an automated visual display of several model fit criteria recorded when fitting multi-GBTM for different and increasing numbers of clusters. For a closer description of this plot, readers are referred to the original publication[1]. Briefly, the F-CAP combines eight goodness-of-fit and model-adequacy criteria in compact graphs for several user stipulated varying k's (i.e., number of classes). The basic idea behind the F-CAP is that the user can see how indices change by increasing the number of latent trajectories. The condensed visual display of their behavior allows the assessment of different criteria side-by-side to reach a well-informed decision.

Figure 4. **Fit-Criteria Assessment Plots (FCAP) for GBMTM class-enumeration**
Akaike's information criterion (AIC), Bayesian information criterion (BIC) and Likelihood

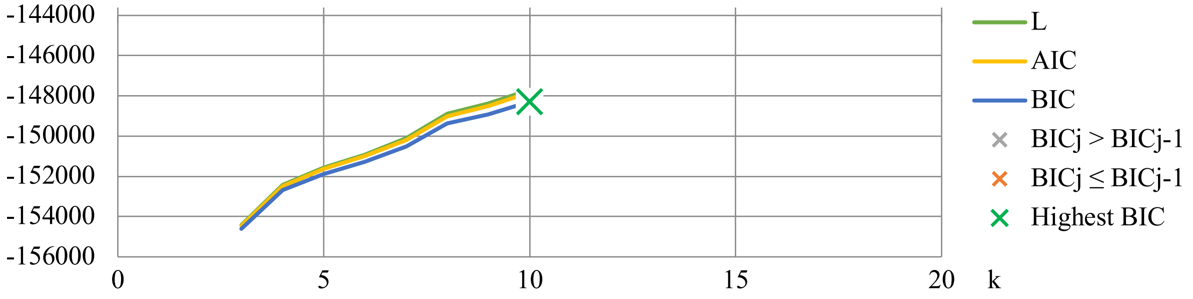

Figure 5. **Average Posterior probability (of assignment), mismatch (between assigned and estimated class probabilities) and SD (of cluster membership probabilities)**

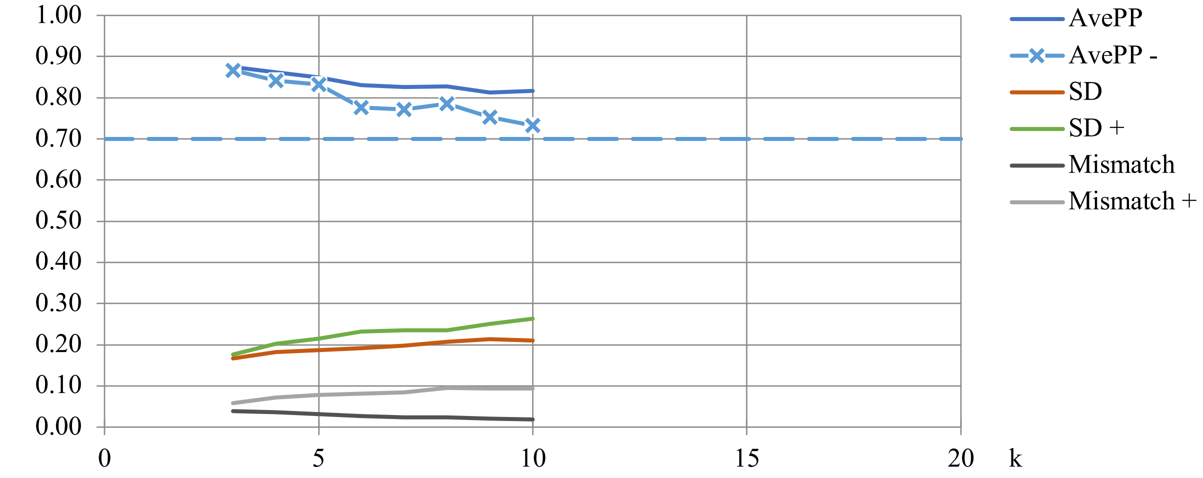

Figure 6. **Odds of correct classification (OCC)**

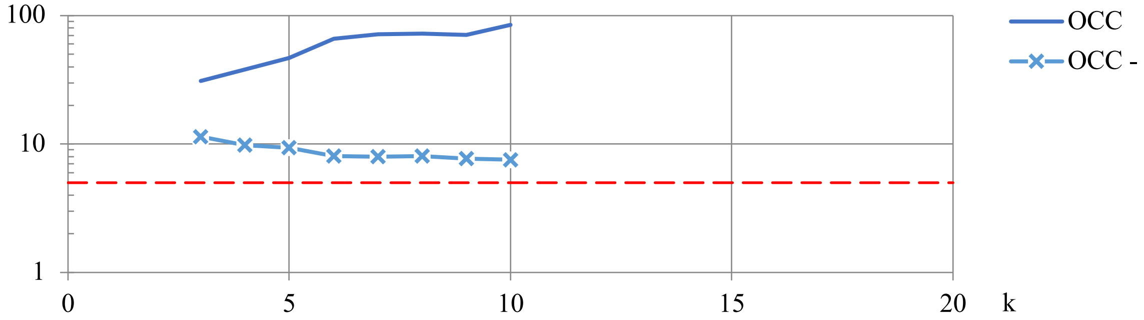

Figure 7. **Percentage of individuals belonging to the smallest cluster**

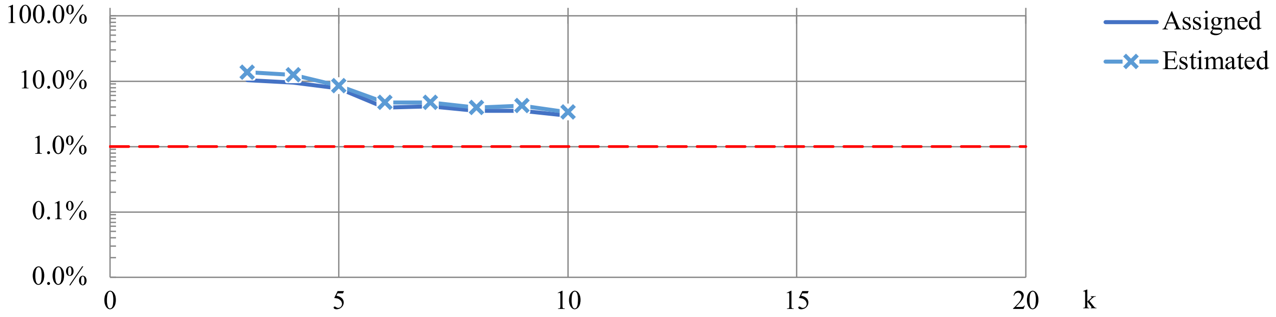

GBMTM - Internalising and Externalising class-enumeration

In our data, the F-CAP showed no clear elbow-bent for any of the likelihood information-based criteria: BIC, AIC and likelihood - top plot – as all of them increased up to the largest attempted *k* attempted. The AvePP, SD, mismatch and OCC criteria (second and third plots) were similarly inconclusive, although in the second plot the average posterior probability of assignment showed minor drops in the transitions from *k*=5 to 6 and 8 to 9 (deterioration of class-assignment quality).

A monotonic improvement of likelihood-based criteria with increasing *k* has been shown to be diagnostic for variance-covariance model under-specification. GBTM does not allow for within-class variability to be modelled via e.g. random effects or the classes to have distinct variance-covariance structure (a limitation linked to applied the SAS software). As a result of

the not modelled within class-variability, classes can be further partitioned to give rise to spurious latent classes (which is manifest in trajectories that are not qualitatively different from each other, e.g. they are parallel, have low class-separation and/or are prohibitively small). Because the likelihood-based criteria are particularly prone to such plateauing behavior, when fitting a GBTM users should be aware that their guidance may lead to an erroneous model choice, i.e. class over-extraction[3]. We paid therefore careful attention to avoid it.

A closer appreciation of the multivariate trajectories for different *ks* confirmed the possibility of over-extraction (see plots below). Thus, we resorted to the auxiliary role of the APPA and selected a few best fit candidate models (k = 5 to 8). The transition from class 5 to 6 unveiled a 'chronic troubled' trajectory that was deemed theoretically relevant. The additional transition from 6 to 7 and further, by contrast, did not add to any qualitatively distinct classes, hinting at parallel partitioning yielding overlapping trajectories. We settled, thus, for the 6-class solution.

1. Klijn, S. L., Weijenberg, M. P., Lemmens, P., van den Brandt, P. A., & Lima Passos, V. (2017). Introducing the fit-criteria assessment plot–A visualisation tool to assist class enumeration in group-based trajectory modelling. Statistical methods in medical research, 26(5), 2424-2436.
2. Heggeseth BC, Jewell NP. How Gaussian mixture models might miss detecting factors that impact growth patterns. Ann Appl Stat.2018; 12(1): 222-245.
3. van der Nest, G., Lima Passos, V., Candel, M. J., & van Breukelen, G. J. (2022). Model fit criteria curve behaviour in class enumeration–a diagnostic tool for model (mis) specification in longitudinal mixture modelling. Journal of Statistical Computation and Simulation, 92(8), 1640-1672

Figure 8. **Multivariate trajectories for distinct *ks*:**

K=5

Internalising

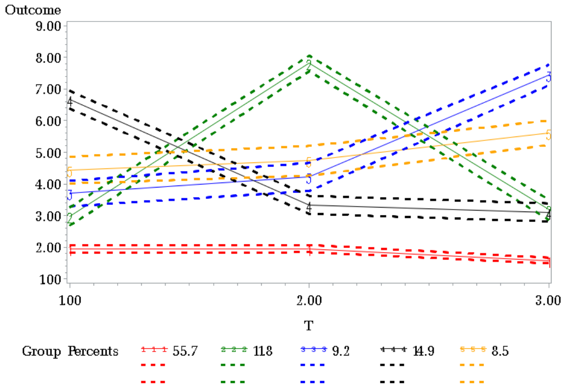

Externalising

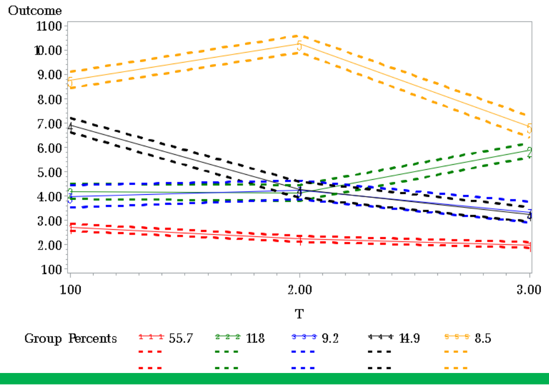

K=6

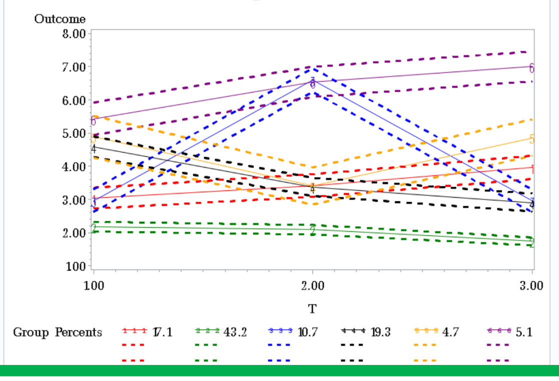

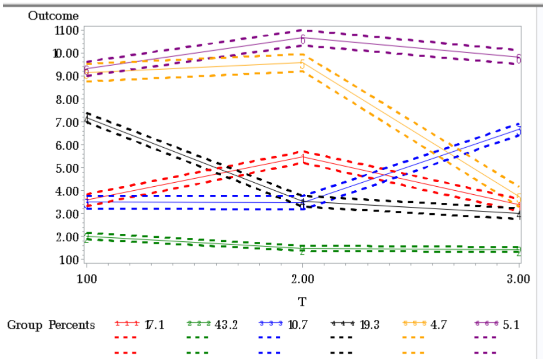

K=7

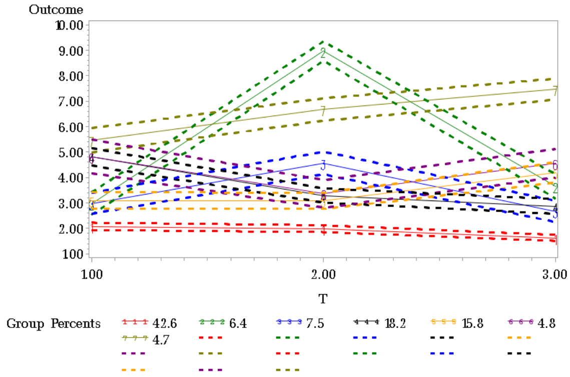

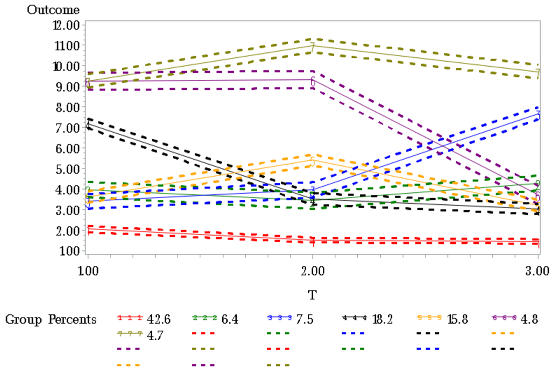

Appendix 3

Association between family profiles and behaviour trajectories

Figure 9: Stacked bar chart, showing the distribution of the LCA's family profiles conditioned on the internalising/externalising joint trajectories

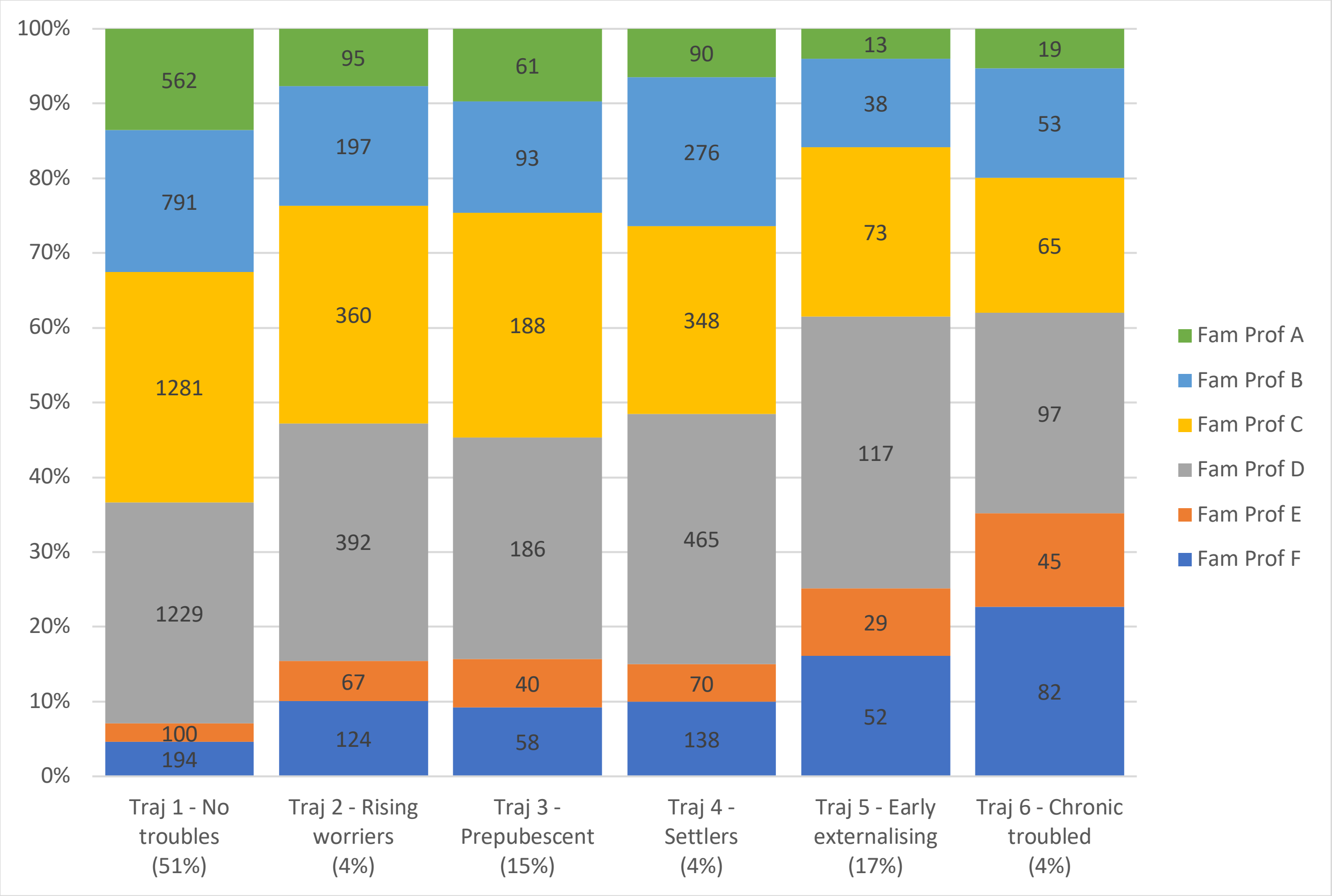

KEY Development Profile Fam Prof A Comfortable (10%); Fam Prof B 'Older mother, low education, low probability of breastfeeding (18%); Fam Prof C 'Older mother, medium maternal education' (29%); Fam Prof D 'Challenged young mother' (31%); `Fam Prof E 'Young mother, low depression, feel unsafe' (4%); Fam Prof F 'Vulnerable'(8%).

Figure 9 shows the unadjusted distributions of the family profiles conditional on the bivariate trajectories of internalising/externalising scores. A gradient pattern is discernible, with the representation of more adverse profiles gradually increasing from Traj 1 (No troubles) to Traj 6 (Chronic troubled).
